# Adult Judges Use Heuristics When Categorizing Infants’ Naturally Occurring Responses to Others’ Emotions

**DOI:** 10.3389/fpsyg.2019.02546

**Published:** 2019-11-14

**Authors:** Peter J. Reschke, Eric A. Walle

**Affiliations:** ^1^School of Family Life, Brigham Young University, Provo, UT, United States; ^2^Psychological Sciences, University of California, Merced, Merced, CA, United States

**Keywords:** emotion, emotion responding, emotion categorization, infant behavior, emotional development

## Abstract

Inferring the motivations of others is a fundamental aspect of social interaction. However, making such inferences about infants can be challenging. This investigation examined adults’ ability to infer the eliciting event of an infant’s behavior and what information adults utilize to make such inferences. In Study 1, adult participants viewed recordings of 24-month-old infants responding to an actor’s emotional display (joy, sadness, fear, anger, or disgust) toward a broken toy and were asked to infer which emotion the actor expressed using only the infant’s behavioral responses. Importantly, videos were blurred and muted to ensure that the only information available regarding the actor’s emotion was the infant’s reaction. Overall, adults were poor judges of the elicitors of infants’ behaviors with accuracy levels below 50%. However, adults’ categorizations appeared systematic, suggesting that they may have used consistently miscategorized emotions. To explore this possibility, a second study was conducted in which a separate sample of adults viewed the original recordings and were asked to identify infants’ goal-directed behaviors (i.e., security seeking, social avoidance, information seeking, prosocial behavior, exploration, relaxed play). Overall, adults perceived a variety of infant differentiated responses to discrete emotions. Furthermore, infants’ goal-directed behaviors were significantly associated with adults’ earlier “miscategorizations.” Infants who responded with specific behaviors were consistently categorized as having responded to specific emotions, such as prosocial behavior in response to sadnesss. Taken together, these results suggest that when explicit emotion information is unavailable, adults may use heuristics of emotional responsiveness to guide their categorizations of emotion elicitors.

Inferring the motivations of others’ behaviors is a fundamental aspect of social interaction. However, making such inferences when observing the behavior of infants can be challenging. This study examined whether adults can infer the eliciting emotional event of an infant’s behavior and what information adults may utilize to make such inferences.

Emotions regulate the behavior of the self and social partners toward adaptive goal-directed responses specific to the emotional context (Campos et al., 1989; Walle and Campos, 2012). For example, an adaptive response to a social partner’s communication of fear is to avoid, rather than engage with, the fear-inducing referent (Sorce et al., 1985; Martin et al., 2008). Recent research indicates that even infants respond with functionally distinct behaviors to adult discrete emotional displays (Walle et al., 2017), suggesting that some differentiation in goal-directed responding may be present, though still developing, prior to other emotionally relevant skills, such as adhering to emotion display rules (see Camras and Shutter, 2010) or labeling emotions (see Widen, 2013). Thus, infants’ functional behavioral responses may be an essential cue for adults when inferring the eliciting events of infants’ behaviors.

One might wonder whether it would be easier to simply assess the infant’s facial expression to infer the eliciting event leading to the behavioral response (Izard, 1979). After all, prior research demonstrates that adults can correctly label children’s emotional expressions (Felleman et al., 1983; though see Oster et al., 1992). However, recent studies indicate a surprising disconnect between children’s emotional states and facial expressions. Specifically, infants do not consistently produce prototypical facial expressions in scenarios commonly associated with specific emotions, and at times even display “atypical” expressions given the context (Camras et al., 2017). Moreover, FACS-trained researchers’ assessments of children’s facial expressions are often incongruent with children’s self-reported emotional experiences (Castro et al., 2018). Thus, while adults have expectations regarding how children should respond in different hypothetical situations, like responding to success with happiness (Zelko et al., 1986; Camras and Allison, 1989), we know of no research that has examined whether adults actively use such assumptions to infer the elicitors of infant behavior.

The above research calls into question whether adults can accurately infer the significant relations that elicit infants’ behaviors—a disconcerting conclusion given the need for caregivers to make such determinations in real-time on a daily basis. Furthermore, it reveals an intriguing paradox: adults possess lay theories regarding young children’s emotional responsiveness, but whether adults can correctly infer the underlying motivation of children’s behavior in emotional contexts is unclear. Thus, this investigation had two primary aims: (1) to investigate whether adults can accurately identify emotional communication eliciting infants’ behaviors based on infants’ behavioral responses to emotions and (2) to explore whether adults’ categorizations are guided by heuristics of emotional responsiveness.

## Study 1

We first examined whether adults could correctly categorize which emotional communication an infant had observed using only the infant’s behavioral response. Previous research indicates that adults have clear behavioral expectations regarding children’s responses in hypothetical emotional situations. Thus, we predicted that adults would demonstrate high accuracy in correctly identifying the specific emotion to which the infant responded.

### Method

#### Participants

A total of 214 undergraduate students (154 female; *M*_age_ = 19.50 years, SD = 1.49) completed the study. The sample was racially diverse, with 102 participants identifying as Hispanic, 58 as Asian, 23 as Caucasian, 13 as African American, 12 as Mixed Race, and 2 as Native Hawaiian or Pacific Islander. Four participants declined to report racial information. Informed consent was obtained from all individual participants included in the study.

#### Stimuli

Videos of infants responding to discrete emotions were taken from a larger video collection used in previous research (Walle et al., 2017). Each video featured a 24-month-old infant situated between her/his caregiver, an actor, and a basket of age-appropriate toys. Once the infant was within reach of the actor, the actor revealed a plush bunny doll that had previously been intact, but now had one leg ripped off with stuffing spilling out. The actor then expressed facially, posturally, and vocally one of five emotions (joy, sadness, fear, anger, disgust) toward the bunny, and the infant was given 45 s to respond. A hidden camera located behind the actor, facing the infant, captured the events and infants’ responses.

A total of 76 videos were used in the present study and included the following number of infants in each condition: joy (*n* = 17), sadness (*n* = 13), fear (*n* = 14), anger (*n* = 18), and disgust (*n* = 14). The discrepancy in the number of videos within each emotion condition was the result of not all families providing consent for their video to be used in the present study, and some infants failing to complete the paradigm in the original study by Walle et al. (2017). Each video featured a distinct infant.

Recordings of infant responses were edited using Adobe Premiere to blur and mute the actor so that only the infants’ behaviors (e.g., manual actions, movements, sounds) were observable. This step was essential to ensure that participants had no information regarding the eliciting event other than the infant’s behavioral response.

#### Procedure

Participants first completed a demographics questionnaire and a question regarding participants’ frequency of direct contact with children. The 76 video stimuli were randomly ordered and separated into blocks consisting of up to 16 videos. Blocks were shown separately to groups of participants (range: 33–41 participants per group) in a campus conference room using a projector and speaker system. Participants were informed that each video would feature an infant responding to an actor who was displaying one of five emotions (joy, sadness, fear, anger, disgust) in response to a broken plush doll, and that all visual and auditory information regarding the actor’s emotional reaction had been edited out. Each video was shown twice in succession. Participants were instructed to wait until after the video had been presented before selecting their answer on a response sheet with the following fixed-ordered choices: joy, sadness, fear, anger, disgust. Participants were given approximately 1 min to mark their response. A 3-min break was provided after every five videos to reduce testing fatigue.

A second researcher monitored participants throughout the session to ensure that participants were attentive to the videos and adhered to the instructions. Two participants were excluded for falling asleep or premature marking of answers. The entire testing session lasted approximately 60 min.

### Results

A full confusion matrix of participants’ emotion categorizations is presented in Table 1. Participants’ *accuracy*, operationalized as correctly identifying the emotion displayed by the actor, was analyzed using a generalized linear mixed model specified with a binomial distribution, a compound symmetry covariance structure, and a logit link. Restricted maximum likelihood (REML) was used in the model. Emotion was included as a within-subjects variable. *Post hoc* comparisons were conducted using a Bonferroni correction (*α* = 0.005). Preliminary analyses revealed that participant gender and frequency of direct contact with children (Median = “once a month,” range = “less than once a year” to “almost daily”) were not related with accuracy; thus, these variables were excluded from subsequent analyses.

**Table 1:** Proportion agreement of emotion categorizations of the elicitors of infants*’ behavioral responses in Study 1*.

	Emotion categorization				
Actual emotion	Joy	Sadness	Fear	Anger	Disgust
Joy	0.32	0.37	0.08	0.09	0.15
Sadness	0.11	0.47	0.14	0.09	0.20
Fear	0.17	0.24	0.21	0.19	0.19
Anger	0.13	0.25	0.17	0.25	0.20
Disgust	0.17	0.19	0.27	0.19	0.18

Results indicated a main effect of emotion, *F*(4, 2,718) = 31.06, *p* < 0.001, $\eta_{p}^{2}$ = 0.04. Pairwise comparisons indicated that participants correctly identified sadness (*M* = 47%) stimuli more than joy (*M* = 32%), *t* = 4.85, *p* < 0.001, CI [0.06, 0.23]; anger (*M* = 24%), *t* = 7.89, *p* < 0.001, CI [0.15, 0.31]; fear (*M* = 21%), *t* = 8.79, *p* < 0.001, CI [0.18, 0.35]; and disgust stimuli (*M* = 18%), *t* = 10.10, *p* < 0.001, CI [0.21, 0.37]. Participants also correctly identified joy stimuli more than anger, *t* = 3.23, *p* = 0.001, CI [0.01, 0.15]; fear, *t* = 4.37, *p* < 0.001, CI [0.04, 0.19]; and disgust stimuli, *t* = 5.71, *p* < 0.001, CI [0.07, 0.22]. Accuracy for anger, fear, and disgust stimuli did not differ, *p*’s ≥ 0.008.

### Discussion

Participants more accurately inferred infants’ behavioral responses to sadness and joy elicitors compared to anger, fear, and disgust elicitors. However, contrary to our predictions, participants overall were largely inaccurate, with no single categorization exceeding 50%. This is in contrast to previous research in which adults more accurately identified emotion elicitors (e.g., 60–85%; Camras and Allison, 1989), though it should be noted that such scenarios were hypothetical.

At least two explanations may explain adults’ poor accuracy. First, participants may have categorized emotions randomly, particularly for the anger, fear, and disgust stimuli, which were near chance levels (range: 18–24%). However, the significant main effect of emotion indicates that participants’ responses, though overall incorrect, were systematic, particularly for the sadness and joy stimuli. Thus, a second alternative explanation is that adults systematically made incorrect inferences, perhaps because infants’ behavioral responses did not match adults’ lay theories of emotional responsiveness. For example, an infant responding to a fear display with approach behaviors (e.g., comforting) may have been misclassified as responding to sadness because the adult heuristic may be that one should respond to sadness with prosocial behavior, whereas one should respond to fear with security seeking (Saarni et al., 2006).

To test this latter explanation, a second study was conducted to examine whether adults’ categorizations of infant goal-directed behaviors varied across discrete emotions, and whether such categorizations were associated with the emotional categorizations of stimuli in Study 1.

## Study 2

Study 2 explored whether adults used heuristics to infer the elicitor of infant behavioral responding. It was predicted that (1) adults would differentially categorize behaviors across emotions, and that (2) these behavioral categorizations would correspond with adults’ emotion categorizations from Study 1.

### Method

#### Participants

A total of 199 undergraduate students (136 female, *M*_age_ = 19.85 years, SD = 2.70) completed Study 2. Participants were racially diverse, with 100 participants identifying as Hispanic, 39 as Asian, 19 as Caucasian, 19 as Mixed Race, 11 as African American, 3 as Native Hawaiian or Pacific Islander, and 2 as Native American or Alaskan Native. Six participants declined to report racial information.

#### Stimuli

The original, unedited recordings of the same infants were included in Study 2.

#### Goal-Directed Behaviors

Categories of infant behavioral responses were derived from proposed functional affective responses (Walle and Campos, 2012). Specifically, six goal-directed behaviors were used to characterize infant behaviors: (1) seek security, (2) social avoidance, (3) information seeking, (4) prosocial behavior, (5) exploration, and (6) relaxed play. Full descriptions of the behaviors are provided in Table 2.

**Table 2:** Descriptions of goal-directed behavioral codes.

Goal	Definition
Security seeking	Infant sought comfort or security
Social avoidance	Infant avoided engaging with the experimenter in any way
Information seeking	Infant sought more information about the situation
Prosocial behavior	Infant tried to help the experimenter in some way
Exploration	Infant handled the stimulus in order to learn more about it
Relaxed play	Infant engaged in a playful manner with experimenter, or behavior seems unaffected by emotional display

#### Procedure

The procedures differed from Study 1 in the following ways. Stimuli were separated into blocks consisting of up to 15 videos, and were shown to separate groups of participants (range: 28–36 participants per group). Participants were informed before each video which emotion the adult would be expressing to ensure that all participants were aware of the correct emotional context. A graduate student researcher trained participants to apply behavioral codes to the videos. Training consisted of reviewing detailed explanations of each code (see Table 2) and observing the researcher code one example video. Participants then completed two additional practice trials and again reviewed the coding with the researcher to ensure full comprehension of the coding scheme. Participants were instructed to code the goal-directed behavior most prominently displayed by the infant using the collection of infant behaviors (e.g., looking to experimenter, looking to parent, looking to stimulus, facial affect, location in room, vocalizations, gestures). Three participants were excluded for sleepiness or inattentiveness.

### Results

We first examined whether adult judgments of infant goal-directed behaviors varied across discrete emotion conditions. Participant classifications of each infant goal-directed behavior were separately analyzed using linear mixed effect models specified with a binomial distribution and a logit link. Restricted maximum likelihood (REML) was used in each model. Emotion was included as a within-subjects variable. *Post hoc* comparisons were conducted using a Bonferroni correction for multiple comparisons (*α* = 0.005). Preliminary analyses revealed that participant gender and frequency of direct contact with children (Median = “once a month,” range = “less than once a year” to “almost daily”) were not related with infant behavior categorizations. Thus, these variables were excluded from subsequent analyses.

#### Security Seeking

Results indicated a significant effect of emotion, *F*(4, 2,526) = 31.86, *p* < 0.001, $\eta_{p}^{2}$ = 0.05. Pairwise comparisons indicated that adults identified infant security seeking significantly more in disgust (*M* = 26%), anger (*M* = 25%), and fear (*M* = 24%) stimuli than sadness (*M* = 6%) and joy (*M* = 6%) stimuli (all *p*’s < 0.001). No other significant differences between emotion conditions were present (*p*’s > 0.58).

#### Social Avoidance

A significant effect of emotion was present, *F*(4, 2,526) = 24.27, *p* < 0.001, $\eta_{p}^{2}$ = 0.04, and subsequent comparisons indicated that adults identified infant social avoidance significantly more in fear (*M* = 26%), disgust (*M* = 23%), and anger (*M* = 20%) stimuli than sadness (*M* = 8%) and joy (*M* = 7%) stimuli (all *p*’s < 0.001). There were no other significant differences between emotion conditions (*p*’s > 0.01).

#### Information Seeking

Analyses did not find a significant effect of emotion, *F*(4, 2,526) =2.27, *p* = 0.06, $\eta_{p}^{2}$ = 0.004. Thus, no pairwise comparisons were conducted.

#### Prosocial Behavior

Coding of infant prosocial behavior varied significantly as a function of emotion, *F*(4, 2,526) = 40.56, *p* < 0.001, $\eta_{p}^{2}$ = 0.06. Pairwise comparisons indicated that adults identified infant prosocial behavior significantly more in sadness (*M* = 46%) and joy (*M* = 36%) stimuli than anger (*M* = 20%), fear (*M* = 19%), and disgust (*M* = 14%) stimuli (all *p*’s < 0.001). Differences in prosocial behavior between sadness and joy stimuli were significant (*p* = 0.002). No other significant differences between emotion conditions were present (*p*’s > 0.02).

#### Exploration

Results indicated a significant effect of emotion, *F*(4, 2,526) = 24.59, *p* < 0.001, $\eta_{p}^{2}$ = 0.04. Subsequent comparisons indicated that adults identified infant exploration significantly more in sadness (*M* = 21%) and joy (*M* = 28%) stimuli than anger (*M* = 10%) and fear (*M* = 8%) stimuli (all *p*’s < 0.001). Adult exploration classifications for disgust stimuli (*M* = 15%) were significantly more prevalent than fear and significantly less prevalent than joy stimuli (*p*’s ≤ 0.001). Differences between the remaining emotion conditions were not significant (*p*’s > 0.01).

#### Relaxed Play

Results indicated a significant effect of emotion, *F*(4, 2,526) = 7.29, *p* < 0.001, $\eta_{p}^{2}$ = 0.01. Pairwise comparisons indicated that adults identified infant relaxed play significantly more in joy (*M* = 9%) videos than fear (*M* = 4%) and sadness (1%) videos (*p*’s < 0.001). Additionally, adults’ relaxed play classifications for anger (*M* = 5%), disgust (*M* = 5%), and fear videos were significantly more prevalent than sadness (*p*’s ≤ 0.001). There were no other significant differences between emotion conditions (*p*’s > 0.01).

#### Infant Goal-Directed Behaviors and Adult Emotion Categorizations

We next examined the possibility that infants’ behaviors were associated with adults’ emotion inferences from Study 1. Bivariate correlations revealed several significant associations (see Table 3). Infants categorized as responding to joy were positively associated with relaxed play and exploration, and negatively associated with security seeking. Sadness categorizations were highly correlated with prosocial behavior and exploration, and negatively correlated with security seeking and social avoidance. Infants labeled as responding to fear were associated with increased levels of security seeking and social avoidance and decreased concentrations of prosocial behavior, exploration, and relaxed play. Anger categorizations were associated with high levels of security seeking and social avoidance as well as low levels of prosocial behavior and exploration. Infants characterized as responding to disgust were positively correlated with social avoidance and negatively associated with exploration.

**Table 3:** Bivariate correlations of proportions of goal-directed behavior ratings and emotion categorizations.

Goal-directed behavior						
Emotion categorization	Security seeking	Social avoidance	Information seeking	Prosocial behavior	Exploration	Relaxed play
Joy	−0.29^*^	−0.15	−0.05	0.05	0.32^**^	0.42^**^
Sadness	−0.48^**^	−0.34**	0.18	0.53^**^	0.26^*^	−0.16
Fear	0.76^**^	0.27^*^	−0.19	−0.44^**^	−0.44^**^	−0.28^*^
Anger	0.55^**^	0.26^*^	−0.17	−0.39^**^	−0.31^**^	−0.12
Disgust	−0.02	0.32^**^	0.17	−0.15	−0.25^*^	−0.04

* *p < 0.05*;

** *p < 0.01*.

### Discussion

Study 2 provides evidence that non-expert adults view infants as engaging in differentiated behavioral responses to discrete emotions. In particular, infants were categorized as responding with prosocial behavior most often in the sadness condition, and relaxed play most often in the joy condition. Although other goal-directed behaviors were less differentiated between emotions, they did differ systematically between prototypically “avoid” type emotions (anger, fear, disgust) and “approach” type emotions (joy, sadness; Walle and Campos, 2012). For instance, adults categorized infants as responding with security seeking and social avoidance most in the anger, fear, and disgust conditions, and exploration most in the sadness and joy conditions. Comparison of these results with findings from previous studies is provided in the section “General Discussion.”

Interestingly, infants’ goal-directed behaviors were associated with adults’ emotional inferences from Study 1, supporting a possibility that adults guided their emotional inferences using heuristics about responding to emotions. For example, when observing an infant display prosocial behavior, regardless of the experimenter’s emotion, adults were more likely to categorize the infant as having responded to sadness than fear or anger. Additionally, adults categorized infants engaging in relaxed play as responding to joy more than all other emotions. It is possible that adults’ folk psychology presumes a strict correspondence between specific events and behavioral responses to those events (Frijda et al., 1989; Saarni et al., 2006). Alternatively, adults may have used a more flexible heuristic to categorize infants’ responses, but appeared to be inaccurate due to infant still-developing behavioral responses to discrete emotions (see Walle et al., 2017). These two possible explanations are further elaborated upon below.

## General Discussion

Inferring the eliciting events of infants’ behavior is a complex process involving the interaction of multiple processes. Of particular interest in the present investigation is how adults infer the elicitors of infants’ behavior. Although adults appeared inaccurate in categorizing the elicitor of infants’ behavioral responses to emotions, adult “miscategorizations” varied systematically as a function of infant goal-directed behaviors. This suggests that adults used a heuristic to categorize the elicitor of infants’ behavior. For example, infants demonstrating security seeking were more likely to be categorized as responding to fear or anger than joy or sadness, regardless of the emotion the infant actually observed. Thus, this pattern of findings suggests that adults attempted to perceive the significant relation between the infant and the environment, but were unable to do so accurately.

At least two theoretical explanations hold important implications for adult lay understanding of infant behavior. First, it is possible that adults view specific behaviors as diagnostic for specific emotional responses, such as seeking security in response to anger or fear, or engaging in relaxed play in response to joy. Indeed, emotion researchers have historically sought similar 1:1 mappings for discrete emotions, such as a physiological response (Levenson, 1992) or an appraisal pattern (Roseman, 1984). However, emotion responding is an equipotential process in which multiple behaviors can be adaptive for a given emotional context (Campos et al., 2004). Thus, such rigidity in appreciating goal-directed behavior could be maladaptive in interpersonal contexts, where constant variation of context and relational significance necessitates flexibility in evaluating and responding. It is also possible that the forced-choice design may have suggested that participants apply a rigid heuristic. Additional research using an open-response format would be necessary to rule out this possibility.

Alternatively, adults may possess valid heuristics of emotional responsiveness, but appear “inaccurate” due to infants’ under-developed responses to discrete emotions. Thus, while previous research indicates that infants engage in increasingly differentiated behaviors in response to discrete emotions (Walle and Campos, 2012), such differentiation is likely still developing and may hamper adults’ abilities to infer the eliciting event. Closer examination of developmental trajectories of infant responding to discrete emotions and adults’ interpretations of such responses is needed to clarify such explanations.

## Further Considerations

These findings provide important considerations for research examining infant and adult behavioral responding in relational contexts. First, these results complement previous work examining adults’ emotional judgments of hypothetical situations (e.g., Zelko et al., 1986; Camras and Allison, 1989), as well as recent studies indicating that children often do not display emotions matching their emotional state (Camras et al., 2017). However, further research of adult judgments of infant behavioral responses in additional contexts is needed to examine consistency in such findings.

Additionally, just as infants are likely still developing differentiated behavioral responses, adult heuristics for interpreting infants’ behaviors likely vary across individuals due to differences in past experience, anticipation, and observational learning. This investigation indicates that non-expert adults with relatively infrequent experience with children perceive a variety of infant goal-directed behaviors in response to discrete emotions, which supports previous research using expert judges (Walle et al., 2017) and is consistent with theoretical work relating to functionalist emotion theory (Saarni et al., 2006). Future research comparing caregiver judgments of their own child’s goal-directed behaviors with judgments from non-caregiver adults could address how experience interacting with infants facilitates such judgments. The present sample of undergraduate students reported relatively little experience interacting with infants, which may have precluded any meaningful interpretation of this individual difference measure. Furthermore, studying how parents encourage infant behavioral responses in real-time (e.g., Hornik and Gunnar, 1988; Castro et al., 2015) or recall interpersonal contexts (e.g., Beeghly et al., 1986; Lagattuta and Wellman, 2002) could illuminate how such behaviors are socialized and refined across development.

## Data Availability Statement

The datasets generated for this study are available on request to the corresponding author.

## Ethics Statement

The studies involving human participants were reviewed and approved by UC Merced Institutional Review Board. The patients/participants provided their written informed consent to participate in this study.

## Author Contributions

PR designed the study, collected the data, and analyzed the data. PR and EW wrote the manuscript.

### Conflict of Interest

The authors declare that the research was conducted in the absence of any commercial or financial relationships that could be construed as a potential conflict of interest.
